# Metal‐to‐Semiconductor Transition and Electronic Dimensionality Reduction of Ca_2_N Electride under Pressure

**DOI:** 10.1002/advs.201800666

**Published:** 2018-09-01

**Authors:** Hu Tang, Biao Wan, Bo Gao, Yoshinori Muraba, Qin Qin, Bingmin Yan, Peng Chen, Qingyang Hu, Dongzhou Zhang, Lailei Wu, Mingzhi Wang, Hong Xiao, Huiyang Gou, Faming Gao, Ho‐kwang Mao, Hideo Hosono

**Affiliations:** ^1^ Center for High Pressure Science and Technology Advanced Research Beijing 100094 China; ^2^ Key Laboratory of Metastable Materials Science and Technology College of Material Science and Engineering Yanshan University Qinhuangdao 066004 China; ^3^ Materials Research Center for Element Strategy Tokyo Institute of Technology 4259 Nagatsuta‐cho, Midori‐ku Yokohama Kanagawa 226‐8503 Japan; ^4^ Laboratory for Materials and Structures Institute of Innovative Research Tokyo Institute of Technology Mailbox R3‐4, 4259 Nagatsuta‐cho, Midori‐ku Yokohama 226‐8503 Japan; ^5^ Hawai'i Institute of Geophysics and Planetology School of Ocean and Earth Science and Technology University of Hawai'i at Manoa Honolulu Hawaii 96822 USA; ^6^ Key Laboratory of Applied Chemistry College of Environmental and Chemical Engineering Yanshan University Qinhuangdao 066004 China; ^7^ Geophysical Laboratory Carnegie Institution of Washington 5251 Broad Branch Road NW Washington DC 20015 USA

**Keywords:** electrides, electronic dimensionality, metal‐to‐insulator transition, phase evolution

## Abstract

The discovery of electrides, in particular, inorganic electrides where electrons substitute anions, has inspired striking interests in the systems that exhibit unusual electronic and catalytic properties. So far, however, the experimental studies of such systems are largely restricted to ambient conditions, unable to understand their interactions between electron localizations and geometrical modifications under external stimuli, e.g., pressure. Here, pressure‐induced structural and electronic evolutions of Ca_2_N by in situ synchrotron X‐ray diffraction and electrical resistance measurements, and density functional theory calculations with particle swarm optimization algorithms are reported. Experiments and computation are combined to reveal that under compression, Ca_2_N undergoes structural transforms from *R* $\bar{3}$ *m* symmetry to *I* $\bar{4}$2*d* phase via an intermediate *Fd* $\bar{3}$ *m* phase, and then to *Cc* phase, accompanied by the reductions of electronic dimensionality from 2D, 1D to 0D. Electrical resistance measurements support a metal‐to‐semiconductor transition in Ca_2_N because of the reorganizations of confined electrons under pressure, also validated by the calculation. The results demonstrate unexplored experimental evidence for a pressure‐induced metal‐to‐semiconductor switching in Ca_2_N and offer a possible strategy for producing new electrides under moderate pressure.

## 1 Introduction

Electrides are stoichiometric ionic solids in which trapped electrons serve as the anions. The loosely bound anionic electrons are independent of any particular atoms or molecular in the lattices. Since first crystalline organic electride of Cs^+^(18‐crown‐6)_2_e^−^ was synthesized by Ahmed et al. in 1983,^1^ many attempts have been made to obtain the stable organic electrides to further understand the nearly‐free electron behaviors and their geometrical topologies. The discovery of room‐temperature stable inorganic electride [Ca_24_Al_28_O_64_]^4+^·4e^−^ (C12A7)^2^ has drawn much attention to electride materials due to the promising practical applications and the substantial amounts of studies have been carried out to find new electrides.^3^ Dicalcium nitride (Ca_2_N) and yttrium hypocarbide (Y_2_C) were demonstrated subsequently as a new class of two‐dimensional (2D) electrides in experiments. Ca_2_N and Y_2_C electrides share the same structural motif: excess electrons are weakly localized in the interlayer spaces between two positively charged [Ca_2_N]^+^ or [Y_2_C]^2+^ layers.[[qv: 3d–g]] Moreover, the recently successful exfoliation of Ca_2_N into monolayer or multilayers greatly expands the technological applications of electrides in batteries,^4^ electronics,[[qv: 3d]] catalysis,^5^ and effective electron donor in chemical reactions.[[qv: 5b,6]] In addition, elemental electrides, including alkaline and alkaline earth metal, are also found to be stabilized at high pressure, named high‐pressure electrides (HPEs).^7^

The intrinsic features of electron localization and delocalized band of anionic electrons revealed in electrides offer the great opportunities for the studies of distinctive electronic properties.^8^ Previous studies on HPEs also show that electrons confinement in electrides usually leads to unprecedented transitions of metal to semiconductor or insulator under pressure.[[qv: 7a,e]] Metal‐to‐semiconductor/insulator transition is fundamental interesting and well‐documented in alkaline metals (e.g., Li,^9^ Na,[[qv: 7a,10]] and K,^11^) and d‐electron systems^12^ under pressure. Pressure is one of the controllable thermodynamic parameters that may modify the electronic structures of materials. On the fundamental level, studies of pressure effects on electrides are of particular interest. Indeed, a high‐pressure X‐ray diffraction investigation for Ca_2_N up to 14.4 GPa suggested a structural transition to a possible distorted anti‐Th_3_P_4_‐type structure (space group: *I* $\bar{4}$3*d*).^13^ Recently, theoretical prediction identified a tetragonal *I* $\bar{4}$2*d*‐type structure as the high‐pressure phase and also found a demetallization process of Ca_2_N under pressure.^14^ However, detailed structures of compressed Ca_2_N were not fully resolved on a purely experimental basis. Studies of pressure effects on electrides enable key insights into the dependence of electron localizations and structural topologies. Moreover, it is of particular importance to perform the experimental studies on the electrical characteristics in order to reveal new aspect of possible metal‐to‐semiconductor transition in Ca_2_N. Therefore, here we experimentally demonstrated pressure‐induced transition of Ca_2_N in structural and electrical characteristics by in situ synchrotron X‐ray diffraction (XRD) and electrical resistance measurements in a diamond anvil cell (DAC) at pressures up to 50 GPa, together with supporting density functional theory (DFT) calculations and particle swarm structural searching.

## 2 Results and Discussion

**Figure 1:**
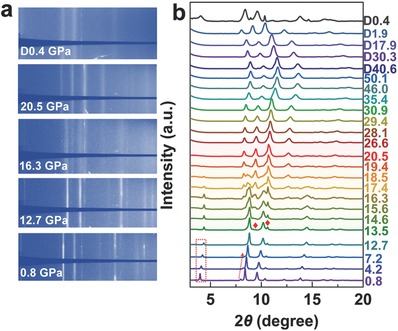
Synchrotron XRD patterns of Ca_2_N obtained under compression up to 50.1 GPa and decompression. a) The raw 2D XRD images at selected pressures during compression and decompression. b) Integrated XRD profiles under different pressures.

Polycrystalline Ca_2_N was prepared by the solid‐state reaction method with Ca_3_N_2_ powder and Ca shots.[[qv: 3d]] Ca_2_N powder was loaded into diamond anvil cell to perform high‐pressure in situ XRD measurements. Collected XRD patterns of Ca_2_N up to 50.1 GPa are shown in **Figure** **1** and Figure S1 (Supporting Information). At 0.8 GPa, the phase can be readily indexed in original *R* $\bar{3}$ *m* symmetry. The obtained lattice parameters with Reitveld refinement show that *c* axis changes rapidly (*a* = 3.60 Å and *c* = 18.76 Å at 0.8 GPa), consistent with previous reports.^13^ With the pressure increasing up to 12.7 GPa, the majority of diffraction patterns remain unchanged, the intensities of (003) and (006) Bragg peaks located at 4.0° and 7.9° at 0.8 GPa decrease significantly and (003) diffraction peak is almost invisible at 7.2 GPa. With further compression to 20.5 GPa, the initial diffraction rings disappear, a complete new phase with four strong rings shows up, together with several weak peaks at high angles. Above 30.9 GPa, the change of relative scattering intensities between Bragg peaks might imply another phase transition up to 50.1 GPa. On decompression, we found the high‐pressure phase of Ca_2_N to recover to original structure at about 0.4 GPa.

**Figure 2:**
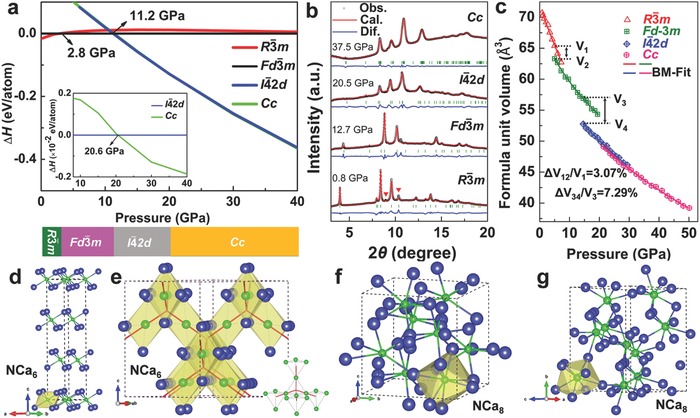
a) The enthalpy per atom for Ca_2_N in *R* $\bar{3}$ *m*, *I* $\bar{4}$2*d*, and *Cc* symmetry as a function of pressure with respect to *Fd* $\bar{3}$ *m*‐type Ca_2_N. Inset: the enthalpy per atom for Ca_2_N in *Cc* symmetry as a function of pressure with respect to *I* $\bar{4}$2*d* type Ca_2_N. b,c) Rietveld refinements on XRD patterns of Ca_2_N under different pressure and the pressure dependence of formula unit volume. The marked peaks around 9° and 10.6° are caused by CaO with *Fm* $\bar{3}$ *m* structure (see Figure S6). d–g) Crystal structures of *R* $\bar{3}$ *m* (d), *Fd* $\bar{3}$ *m* (e), *I* $\bar{4}$2*d* (f), and *Cc* (g) Ca_2_N. The blue and green spheres represent Ca and N atoms, respectively. The red guide lines in *Fd* $\bar{3}$ *m* structure are showing tetrahedral configurations, not bonding.

To address the structure evolutions in experiment, we therefore perform careful structure searching under pressure within the particle swarm optimization algorithm. As displayed in **Figure** **2**a. We first identified a low‐pressure phase transformation from the layer‐like ground state *R* $\bar{3}$ *m* structure to a cubic *Fd* $\bar{3}$ *m* structure at 2.8 GPa. Strikingly, *Fd* $\bar{3}$ *m* structure adopts a 3D framework in which N_3_ units form a tetrahedral configuration (Figure 2e), resemblance to diamond. The structural stability of *Fd* $\bar{3}$ *m* structure was verified by the calculations of elastic constants (*C*_11_ = 64, *C*_44_ = 34 and *C*_12_ = 35 GPa which meets the criteria for mechanical stability) and phonon dispersion (Figure S2, Supporting Information). The simulated XRD pattern of *Fd* $\bar{3}$ *m* structure matches well with the experimental one at 12.7 GPa (Figure S3, Supporting Information). The *R* $\bar{3}$ *m* (Figure 2d) and *Fd* $\bar{3}$ *m* (Figure 2e) structures have identical coordination (NCa_6_) and close bond length of Ca–N (2.43 and 2.45 Å, respectively), explaining the small energy difference and volume collapse (1.8% at 2.8 GPa) between them. With pressure increasing, *Fd* $\bar{3}$ *m* type structure is theoretically stable up to 11.2 GPa. Continuing compression, we successfully reproduce two competitive *I* $\bar{4}$2*d*‐ and *Cc*‐type structures.^14^ In experiment, the high‐pressure phase of Ca_2_N above 20.5 GPa can be well‐fitted by *I* $\bar{4}$2*d* phase (see in Figure S4, Supporting Information), the starting transition pressure is found to be 13.5 GPa, as indicated by the new peaks at ≈9.4° and 10.6° associated with (211), (202), and (103) crystal planes of *I* $\bar{4}$2*d* phase. The observed broadening diffraction peaks above 20.5 GPa can be explained by overlapping doublets peaks of this tetragonal structure. With further compression, however, the tetragonal structure might not be a unique solution given the structural similarity and very tight energetic competition with *Cc*‐type structure. The careful observations found the intensity alternation from second‐strongest peak at 20.5 GPa to the weakest one at 9.4° from 30.9 GPa. Simulated diffraction patterns (Figure S5, Supporting Information) and total energy calculations (Figure 2a) support the transition to *Cc‐*type structure above 30.9 GPa.

Figure 2b shows Reitveld refinements for representative XRD patterns of Ca_2_N taken at 0.8, 12.7, 20.5, and 37.5 GPa using *R* $\bar{3}$ *m*, *Fd* $\bar{3}$ *m*, *I* $\bar{4}$2*d*, and *Cc* structures, respectively. At 12.7 GPa, the lattice constant of *a* = 9.79 Å is obtained using *Fd* $\bar{3}$ *m* structure. At higher pressure, this cubic Ca_2_N transforms to tetragonal *I* $\bar{4}$2*d* structure with lattice parameters of *a* = 7.25 Å and *c* = 7.49 Å at 20.5 GPa, and then to a monoclinic *Cc*‐type with the lattice constants of *a* = 6.90 Å, *b* = 10.06 Å, *c* = 5.94 Å, and β = 124.39° at 37.5 GPa. The Reitveld lattice parameters of these structures are well consistent with our predictions (Table S1, Supporting Information). The pressure‐dependent volume changes (Figure 2b) are fitted by three‐order Birch–Murnaghan equation of state,^15^ we obtained the bulk moduli and volumes per unit of *B*_0_ = 48.9 ± 3.9 GPa and *V*_0_ = 72.1 ± 0.3 Å^3^ for *R* $\bar{3}$ *m* phase, *B*_0_ = 59.7 ± 4.3 GPa and *V*_0_ = 68.2 ± 0.3 Å^3^ for *Fd* $\bar{3}$ *m* phase, *B*_0_ = 65.1 ± 9.6 GPa and *V*_0_ = 63.3 ± 1.1 Å^3^ for *I* $\bar{4}$2*d* phase, and *B*_0_ = 63.7 ± 6.7 GPa and *V*_0_ = 62.7 ± 1.1 Å^3^ for *Cc* phase. At 5 GPa, *R* $\bar{3}$ *m* structure transforms to *Fd* $\bar{3}$ *m* structure with volume collapse (Δ*V*_12_/*V*_1_) of 3.07%. The volume change (Δ*V*_34_/*V*_3_) from *Fd* $\bar{3}$ *m* structure to *I* $\bar{4}$2*d* structure at 14.6 GPa is 7.29%. There is almost no volume collapse from *I* $\bar{4}$2*d* to *Cc* structure.

**Figure 3:**
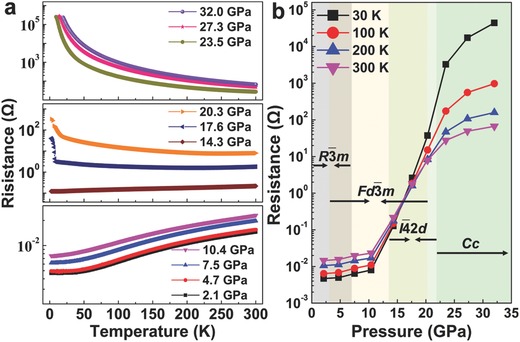
Electrical resistance changes with pressure and temperature. a) Temperature dependence of electrical resistance from 2 to 300 K. The curves obtained under pressures show the temperature dependence of electrical resistance changed from positive to negative. b) Electrical resistance values as a function of applied pressure at different temperature of 30, 100, 200, and 300 K, respectively.

**Figure 4:**
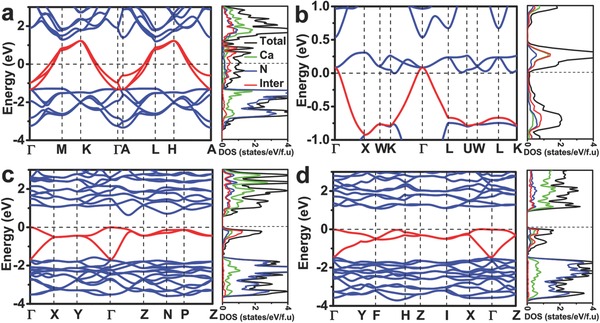
Calculated band structure, total and partial density of states (TDOS and PDOS) for a) *R* $\bar{3}$ *m*, b) *Fd* $\bar{3}$ *m*, c) *I* $\bar{4}$2*d*, and d) *Cc* structures. The red curves in the band structure are the interstitial bands where the anionic electrons occupied mainly. For *Fd* $\bar{3}$ *m* structure, the spin–orbit coupling (SOC) calculations give a band gap of 0.2 meV. *I* $\bar{4}$2*d* and *Cc* were calculated at 11.2 and 20.6 GPa, respectively, the calculated band gap with HSE06 is found to be 1.44 and 2.88 eV, respectively.

It is very interesting to reveal the electronic properties of Ca_2_N phases. Our band structure calculations (Figure 4) reveal a new transformation from initial metal (*R* $\bar{3}$ *m*), semimetal (*Fd* $\bar{3}$ *m*) to semiconductor (*I* $\bar{4}$2*d* and *Cc*) as pressure increased. In *Fd* $\bar{3}$ *m* structure (Figure 4b), the interstitial band shows linear dispersion near the Fermi energy and triply degenerates with conduction bands at the Γ point, which is protected by the crystal symmetry. The semimetal behavior of cubic phase is further supported by the calculations of the spin–orbit coupling and HSE06 hybrid functional. In experiments, the electrical resistance measurements for Ca_2_N are performed from 2 to 300 K at varying pressures. **Figure** **3**a shows the changes of electrical resistance of Ca_2_N under pressure. Below 10.4 GPa, the resistance–temperature slope d*R*/d*T* clearly shows metallic character: a positive temperature dependence. At 14.3 GPa, the resistance is linearly dependent on the temperature and slightly increases with the temperature, showing a clear structural transition. With the increase of pressure, the resistance‐temperature curves have a negative slope, indicating a typical semiconducting character. Generally, high pressure leads to insulator‐to‐metal or semiconductor‐to‐metal transition because of reduction of interatomic distances and strengthening of interatomic interactions.^16^ The observed metal‐to‐semiconductor transition in Ca_2_N should be attributed to the structural transitions starting at 13.5 GPa, in agreement with in situ XRD results and theory by Zhang et al.^14^ (Figure 1b). **Figure** **4**b shows the curves of electrical resistance versus pressure at selected temperatures. Below 10 GPa, the electrical resistance mildly increases with the pressure, caused by modifications of delocalized electrons. The room‐temperature (300 K) electrical resistance is 14.56 mΩ under 0.8 GPa and 15.51 mΩ at 4.7 GPa. However, the resistance‐pressure slope d*R*/d*P* shows uptrend in the pressure range from 4.7 to 7.5 GPa (19.70 mΩ), which should be contributed to phase transition from *R* $\bar{3}$ *m* to *Fd* $\bar{3}$ *m*. The electrical resistance increases significantly to 27.58 Ω when pressure reaches at 23.5 GPa, which is associated with the complete formation of semiconducting *I* $\bar{4}$2*d* and *Cc* phases of Ca_2_N.

**Figure 5:**
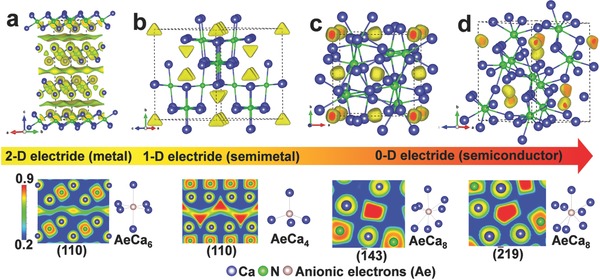
Calculated electron localization functions (ELFs) for a) *R* $\bar{3}$ *m*, b) *Fd* $\bar{3}$ *m*, c) *I* $\bar{4}$2*d*, and d) *Cc* structures with isosurface value 0.55, 0.9, 0.9, and 0.9, respectively. The ELF maps around anionic electrons accompanied with the coordination environment of anionic electrons (Ae) are showed in the bottom. The distribution of anionic electrons reveals a transition from 2D layer (*R* $\bar{3}$ *m*), 1D chain (*Fd* $\bar{3}$ *m*), and 0D spheres (*I* $\bar{4}$2*d* and *Cc*).

To further understand the electride feature and origin of metal‐to‐semiconductor transition of Ca_2_N, the electron localization function (ELF)^17^ has been calculated, together with total and partial density of states (Figure 4). In ELF distributions (**Figure** **5**), the great value of ELF maximum off the nuclei can be observed in Ca*_n_* (*n* = 4, 6, and 8) cages of *R* $\bar{3}$ *m*, *Fd* $\bar{3}$ *m*, *I* $\bar{4}$2*d*, and *Cc* structures, which is a hallmark of electrides. Partial charge density (Figure S7, Supporting Information) suggests that the anionic electrons are occupied mainly around the Fermi level, termed “interstitial bands” (red curves in band structure). Through counting the electrons of the interstitial bands, there are two electrons trapped in Ca*_n_* (*n* = 4, 8) cages of *Fd* $\bar{3}$ *m*, *I* $\bar{4}$2*d*, and *Cc* structures and one electron in Ca_6_ cages of *R* $\bar{3}$ *m* structures. This magnitude of anionic electrons is strongly correlated with the surrounded Ca*_n_* cages. Each Ca*_n_* cage equates with two (in *R* $\bar{3}$ *m* structure (shared by three Ca_6_ cages)) and four (in *Fd* $\bar{3}$ *m* (shared by separate Ca_4_ cage), *I* $\bar{4}$2*d* and *Cc* structures (shared by two Ca_8_ cages) Ca atoms which proportion to the anionic electrons in Ca_n_ cages. Since the Fermi level is dominated by interstitial bands, the distributions and bonding characters of anionic electrons are thus considered to be responsible for the transitions of electronic properties.

Understanding of the interactions between anionic electrons and adjacent atoms is of vital importance for the electronic changes of Ca_2_N under pressure.^18^ As observed in the ELF maps (Figure 5), the bonding between anionic electrons and adjacent Ca atoms are typical ionic for all Ca_2_N structures. In *R* $\bar{3}$ *m* structure, the anionic electrons interact with each other and are dispersed loosely in the interlayer spaces to form a 2D electron gas.^19^ In *Fd* $\bar{3}$ *m* (Figure 5b) structure, the relatively great ELF value between the nearest anionic electrons suggests a strong interaction, which gives rise to a one‐dimensional (1D) electron chain of anionic electrons. The distinctive 2D and 1D electron distributions provide electron conductions in metallic *R* $\bar{3}$ *m* and semimetal *Fd* $\bar{3}$ *m* phases and exhibit the delocalized PDOS profiles of anionic electrons (Figure 4a,b). In contrast with *R* $\bar{3}$ *m* and *Fd* $\bar{3}$ *m* structures, the anionic electrons are well‐localized in Ca_8_ cages for *I* $\bar{4}$2*d* and *Cc* structures. These separations of anionic electrons formed a zero‐dimensional (0D) electron sphere in *I* $\bar{4}$2*d* and *Cc* structures, which leads to a relatively sharp and narrow DOS peak (near 0.4 eV in *I* $\bar{4}$2*d* and 0.4 and 1.0 eV in *Cc*, see Figure S8c,d, Supporting Information). The separations of anionic electrons are also connected with the pressure‐induced changes of coordination environment of Ca atoms (AeCa_8_, Ae = anionic electrons). This 0D electron sphere of anionic electrons in *I* $\bar{4}$2*d* and *Cc* structures explains the semiconducting behavior of Ca_2_N, similar scenario can be also observed in the semiconducting 0D electrides of C12A7^2^ and Na_2_He,^20^ in which the bonding between constituted atoms are typical ionic and the anionic electrons are well‐separated with each other.

## 3 Conclusion

In summary, the structural and electronic evolutions of Ca_2_N up to 50.1 GPa were investigated by in situ synchrotron X‐ray diffraction and electrical resistance measurements, together with DFT calculations. We identified a first phase transition from *R* $\bar{3}$ *m* to *Fd* $\bar{3}$ *m* structures. Furthermore, both the simulated patterns and total energy calculations suggest the transition from *I* $\bar{4}$2*d* to *Cc*‐type structure under relatively high pressure. Electrical resistance measurements give a direct evidence of pressure‐induced metal‐to‐semiconductor transition, which was accompanied with the reduced dimensionality of anionic electrons from 2D, 1D to 0D. Our results provide the first experimental example for the understanding of underlying the correlation among atomic, electronic, and transport properties of electrides under high pressure.

## 4 Experimental Section

*Synthesis of Ca_2_N*: Polycrystalline Ca_2_N was synthesized by solid state reaction of Ca_3_N_2_ powder (99%, Sigma‐Aldrich) and Ca shots (99.99%, Sigma‐Aldrich). All starting materials for the synthesis were prepared in a glove box (Miwa Mfg. Co., Ltd) filled with purified Ar gas (H_2_O, O_2_ <1 ppm). The mixture of starting materials was wrapped in a Mo foil and sealed in a stainless tube with two caps at both ends. This stainless tube was heated at 800 °C for 2 days.

*In Situ High‐Pressure Characterizations*: High pressure was generated by using a symmetrical DAC with 300 µm culet. Polycrystalline Ca_2_N and a ruby sphere (for pressure calibration) were loaded into the sample chamber (120 µm in diameter) in the center of the preindented Rhenium gasket, but no pressure medium was used to avoid contaminations because of the extremely high reactivity of Ca_2_N. The in situ high pressure XRD patterns with a wavelength of 0.4337 Å were collected at 13‐BMC of the Advanced Phonon Source, Argonne National Laboratory.^21^ The compression and decompression experiments were conducted at room‐temperature. The 2D diffraction patterns were integrated into 1D profile with the Dioptas program.^22^ Structure refinements were performed by GSAS program.^23^

The high‐pressure electrical resistance measurements were performed in a screw‐pressure‐type DAC made of non‐magnetic Be–Cu alloy. The diamond culet was 300 µm in diameter. The Be–Cu gasket was preindented to 10 GPa in a DAC, and then a hole of 270 was drilled in the center of the preindented gasket. The cubic boron nitride powders were added inside the gasket, which were then pressed to 25 GPa, reliably insulating the sample and electrodes against the Be–Cu gasket. Finally, a hole of 140 µm was drilled in the center of the gasket using laser drilling. Four hand‐cut platinum foil strips of 4 µm thickness were directly attached to the sample by using van der Pauw topology as electrodes under a microscope. The pressure was calibrated by using the ruby fluorescence shift at room temperature.

*First‐Principles Calculations*: The structure search was performed using the particle swarm optimization methodology as implemented in the CALYPSO code.^24^ The pressures of structure search were set at 0, 20, 50, and 100 GPa and the simulation cells containing up to eight units. Structure relaxations and electronic structure calculations were performed using DFT within the generalized gradient approximation in the Perdew–Burke–Ernzerhoff functional^25^ as implemented in the Vienna ab initio simulation package VASP code.^26^ The projector augmented wave method^27^ for ionic potentials was adopted, where the valence states are treated as 3s^2^3p^6^4s^2^ and 2s^2^2p^3^ for Ca and N atoms, respectively. A plane wave basis set with energy cutoff of 600 eV and a dense K‐point grid with the spacing of 2π × 0.03 Å^−1^ was used to make sure the convergence of total energies less than 1 meV per atom. The single‐point energy calculations using a hybrid functional of HSE06^28^ were also carried out to estimate the band gap of *I* $\bar{4}$2*d* and *Cc* structures. Phonon calculations were performed in the PHONOPY code.^29^

## Conflict of Interest

The authors declare no conflict of interest.

## Supporting information

Supplementary
