# Supplementary material for: Metal‐to‐Semiconductor Transition and Electronic Dimensionality Reduction of Ca2N Electride under Pressure
Source: Adv Sci (Weinh). 2018 Sep 1;5(11):1800666. doi: 10.1002/advs.201800666 (PMC6247025; doi:10.1002/advs.201800666)
Supplement: Supplementary file 1 — Supplementary [file ADVS-5-1800666-s001.pdf]

Copyright WILEY-VCH Verlag GmbH & Co. KGaA, 69469 Weinheim, Germany, 2018.

ADVANCED SCIENCE
Open Access

Supporting Information

for *Adv. Sci.,* DOI: 10.1002/advs.201800666

Metal-to-Semiconductor Transition and Electronic Dimensionality Reduction of Ca_2N Electride under Pressure

*Hu Tang, Biao Wan, Bo Gao, Yoshinori Muraba, Qin Qin, Bingmin Yan, Peng Chen, Qingyang Hu, Dongzhou Zhang, Lailei Wu, Mingzhi Wang, Hong Xiao, Huiyang Gou, Faming Gao, Ho-kwang Mao, and Hideo Hosono**

Supporting information for

Metal-to-Semiconductor Transition and Electronic Dimensionality Reduction of Ca_2N Electride under Pressure

Hu Tang,[a,b,+] Biao Wan,[a,b,+] Bo Gao,[a,+] Yoshinori Muraba,[c,d] Qin Qin,[a] Bingmin Yan,[a] Peng Chen,[b] Qingyang Hu,[a] Dongzhou Zhang,[e] Lailei Wu,[b] Mingzhi Wang,[b] Hong Xiao,[a] Huiyang Gou,[*,a,f] Faming Gao,[f] Ho-kwang Mao,[a,g] Hideo Hosono[*,c,d]

[a]Center for High Pressure Science and Technology Advanced Research, Beijing 100094, China

[b]Key Laboratory of Metastable Materials Science and Technology, College of Material Science and Engineering, Yanshan University, Qinhuangdao 066004, China

[c]Materials Research Center for Element Strategy, Tokyo Institute of Technology, 4259 Nagatsuta-cho, Midori-ku, Yokohama, Kanagawa 226-8503, Japan

[d]Laboratory for Materials and Structures, Institute of Innovative Research, Tokyo Institute of Technology, Mailbox R3-4, 4259 Nagatsuta-cho, Midori-ku, Yokohama 226-8503, Japan

[e]Hawai'i Institute of Geophysics and Planetology, School of Ocean and Earth Science and Technology, University of Hawai'i at Manoa, Honolulu, Hawaii 96822, USA

[f]Key Laboratory of Applied Chemistry, College of Environmental and Chemical Engineering, Yanshan University, Qinhuangdao 066004, China

[g]Geophysical Laboratory, Carnegie Institution of Washington, 5251 Broad Branch Road NW, Washington, DC 20015, USA

[*]Corresponding Authors: huiyang.gou@gmail.com; hosono@msl.titech.ac.jp

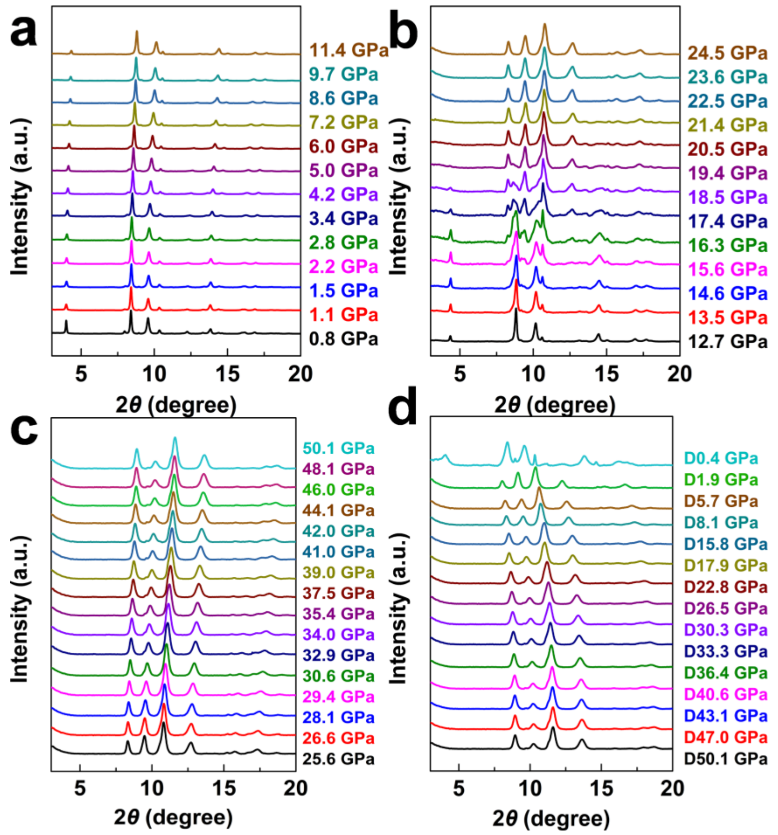

Figure S1: Integrated 1D XRD profiles under different pressure: (a) compressed from 0.8 GPa to 11.4 GPa; (b) from 12.7 GPa to 24.5 GPa; (c) from 25.6 GPa to 50.1 GPa and (d) decompressed from 50.1 GPa to 0.4 GPa.

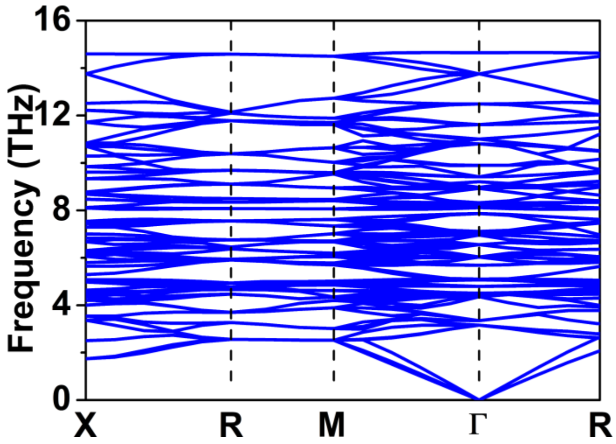

Figure S2: Calculated phonon spectrum for Ca_2N in $Fd\bar{3}m$ symmetry, the absence of imaginary modes suggest the dynamic stability.

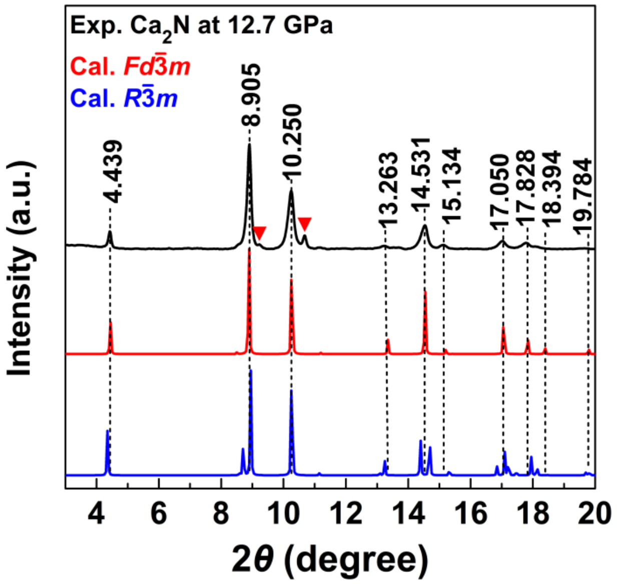

Figure S3: Experimental XRD pattern under 12.7 GPa and calculated XRD patterns of Ca_2N with $R\bar{3}m$ and $Fd\bar{3}m$ structures. The main broad peaks on experiment are indicated by the dashed lines. The $Fd\bar{3}m$ structure was validated by the simulated X-ray diffraction (XRD) patterns and the experimental data. Red triangles indicate the (111) and (200) diffraction peaks of CaO, respectively.

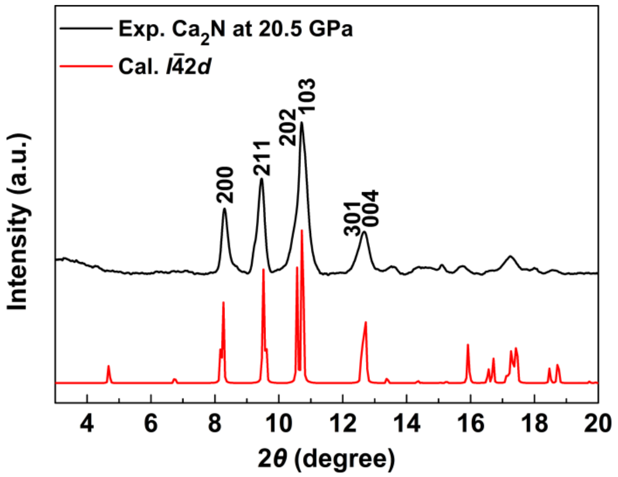

Figure S4: Experimental XRD pattern under 20.5 GPa and calculated XRD pattern of Ca_2N with $I\bar{4}2d$ structure.

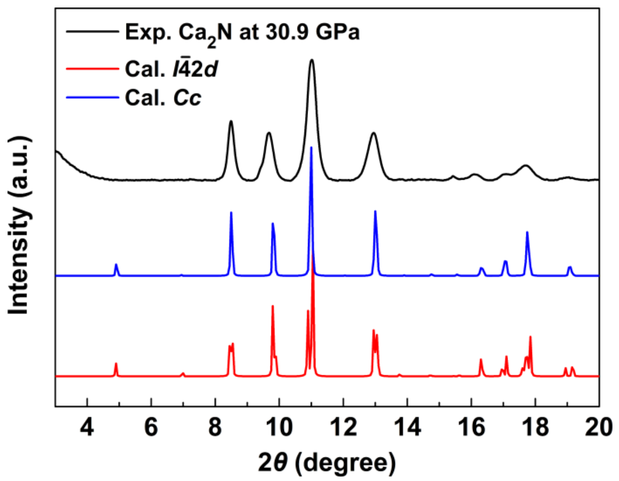

Figure S5: Experimental XRD pattern under 30.9 GPa and calculated XRD patterns of Ca_2N with $I\bar{4}2d$ and *Cc* structures.

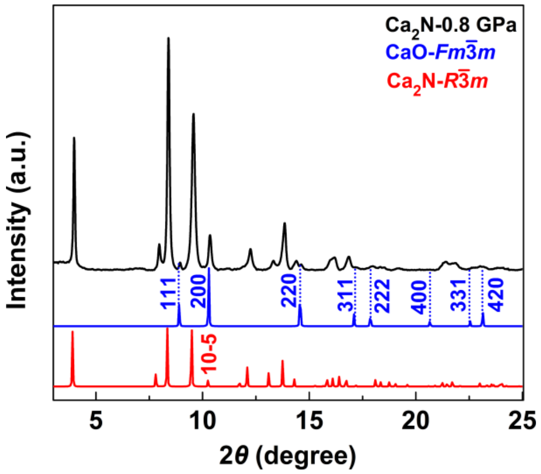

Figure S6: Experimental XRD pattern under 0.8 GPa and calculated XRD patterns of Ca_2N with $R\bar{3}m$ structure and CaO with $Fm\bar{3}m$ structure.

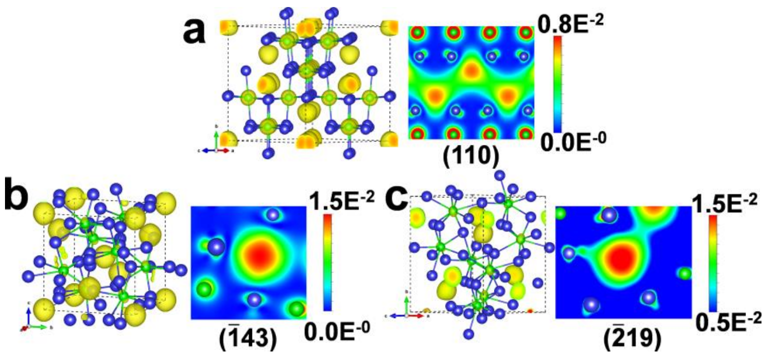

Figure S7: Calculated partial charge density through integration over all K-points of interstitial bands for high pressure Ca_2N phase in $Fd\bar{3}m$ (a), $I\bar{4}2d$ (b) and *Cc* (c) structures. The isosurface value were set as 0.006 $bohr^{-1}$ in Fd-3m and 0.012 $bohr^{-1}$ in $I\bar{4}2d$ (b) and *Cc* (c) structures respectively.

Table S1: Calculated lattice parameters and atomic positions in $Fd\bar{3}m$, $I\bar{4}2d$ and Cc structures at 12.7, 20.5 and 37.5 GPa, respectively. The derived lattice parameters are well consistent with the Reitveld lattice parameters of the structures (with difference ranging from 0.17% to 3.47% at same pressure).

Space group	Lattice parameter (Å)	Atom	site	x	y	z
$Fd\bar{3}m$	a = 9.69	Ca	32e	0.6155	0.3845	0.6155
		N	16d	0.1250	0.1250	0.6250
$I\bar{4}2d$	a = 7.12	Ca	16e	0.3114	0.5658	0.6806
	c = 7.23	N	8d	0.7500	0.1314	0.8750
Cc	a = 6.86	Ca	4a	0.4662	0.0637	0.9026
	b = 9.73	Ca	4a	0.0298	0.0054	0.2610
	c = 5.93	Ca	4a	0.7144	0.1790	0.4007
	β= 124.91	Ca	4a	0.4163	0.2470	0.5272
		N	4a	0.2790	0.8745	0.6404
		N	4a	0.7226	0.9361	0.2757
